# Nasal Reconstruction Using Free TAPAS Flap for Internal Lining Restoration after Hemi-Rhinectomy: A Case Report

**DOI:** 10.1055/a-2870-8089

**Published:** 2026-07-08

**Authors:** Leonardo Brambilla, Giorgio Giacomini, Arianna Gatto, Erica Michela Cavalli, Andrea Marchesi

**Affiliations:** 1 Department of Plastic, Reconstructive and Hand Surgery 9265 IRCCS San Gerardo dei Tintori Foundation HospitalMonzaLombardyItaly; 2 Department of Plastic Reconstructive and Aesthetic Surgery 9311 Università degli Studi di Roma La SapienzaRomeRMItaly

**Keywords:** TAPAS flap, nose reconstruction, internal lining

## Abstract

Nasal reconstruction represents a challenge for the three-dimensional structure with a three-layer structure. Internal lining remains the most demanding tissue to be reconstructed. We utilized the free temporal artery posterior auricular skin (TAPAS) flap for reconstructing the internal nasal lining of the vault and septum of a single coana following hemi-rhinectomy. A case study of a 79-year-old male patient with a malignant melanoma illustrates the effectiveness of this approach. A free TAPAS flap was harvested to restore the lining, together with a homolateral forehead flap for the external skin resurfacing. Cartilage was raised from the septum and auricular concha to recreate the nose framework. Although it is relevant, lining reconstruction remains underestimated, especially when approaching hemi-nasal defects. Our technique allows for one-stage reconstruction through the usage of the TAPAS flap, a thin and pliable tissue with a negligible aesthetic impact. The features of the TAPAS flap match the necessities of lining reconstruction of complex nasal defects.

## Introduction

Nasal reconstruction represents a challenge, especially in through-and-through defects. Difficulty lies in the three-dimensional anatomical structure that involves at least three layers: Internal lining, osteocartilaginous framework, and skin envelope, which have to achieve a satisfactory and functional result. 1 2 3 4 Internal lining remains the most demanding and difficult tissue to be reconstructed, although being both the most important and neglected by reconstructive surgeons. 5 Lining covers the raw surface of the external skin flap, preventing late contraction, which may lead to distortion, alar retraction, and vestibular stenosis. It has to be pliable to host the restored framework, avoiding its exposure and infection, while permitting airway flow. 6

For limited defects of internal lining, involving less than half of the nose, various options are available. 6 7 Skin or mucosal grafts can be prone to contraction or failure. 8 Local flaps are useful, but limited by their pivotal movement. 9 The recruitment of the remaining lining, through mucoperichondrial septal flaps, offers a limited amount of tissue. 8

To restore the internal lining of a double-coanal defect, the most suitable options are free flaps or forehead ones. 6 10 11 12

Many free flaps are reported in the literature for total or subtotal lining reconstruction, especially the radial forearm flap. 2 4 13 14 15

The reconstruction of a single-coanal defect of the lining is a major concern. The challenge lies in a balance between utilizing local options and the need for adequate tissue amount for internal resurfacing, which may require the use of a free flap or forehead flap.

We describe a novel technique that allows for the reconstruction of the lining of the vault and septum of a single coana. The temporal artery posterior auricular skin (TAPAS) flap, first described in 2016 by Lassus et al, 16 17 provides a fasciocutaneous flap that is harvested from the posterior auricular region, nourished by the superior branch of the superficial temporal artery ( Fig. 1 ). This flap has been employed, in pedicled or free fashion, to reconstruct facial, oral, and nasal defects. 18 19 A brief review of the literature available on the TAPAS flap is included in this work.

**Fig. 1:**
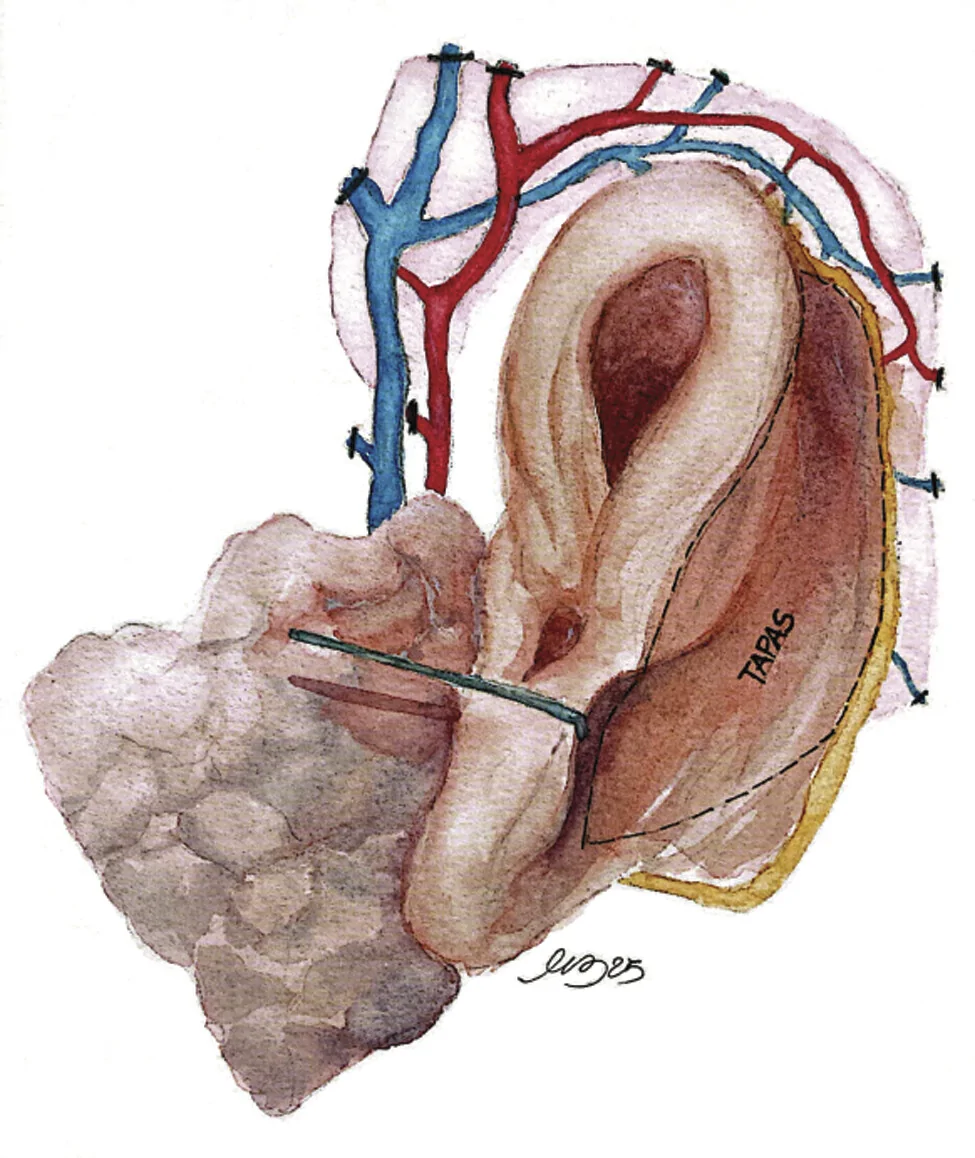
Illustration of the relevant anatomy of the TAPAS flap. TAPAS, temporal artery posterior auricular skin.

The intrinsic features of pliability, softness, and thinness of this little-known flap match the necessities for an optimal resurfacing of the nose lining.

To our knowledge, this is the first work reporting the employment of a free TAPAS flap for the reconstruction of the internal nasal lining, following a hemi-rhinectomy, in a complete three-layer reconstruction.

## Case

A 79-year-old male patient underwent a left hemi-rhinectomy together with an intraparotid sentinel lymph node biopsy for a pT4b N1a malignant melanoma ( Fig. 2 ). Reconstructive time was delayed. No distant metastasis was revealed by clinical examination and instrumental exams.

**Fig. 2:**
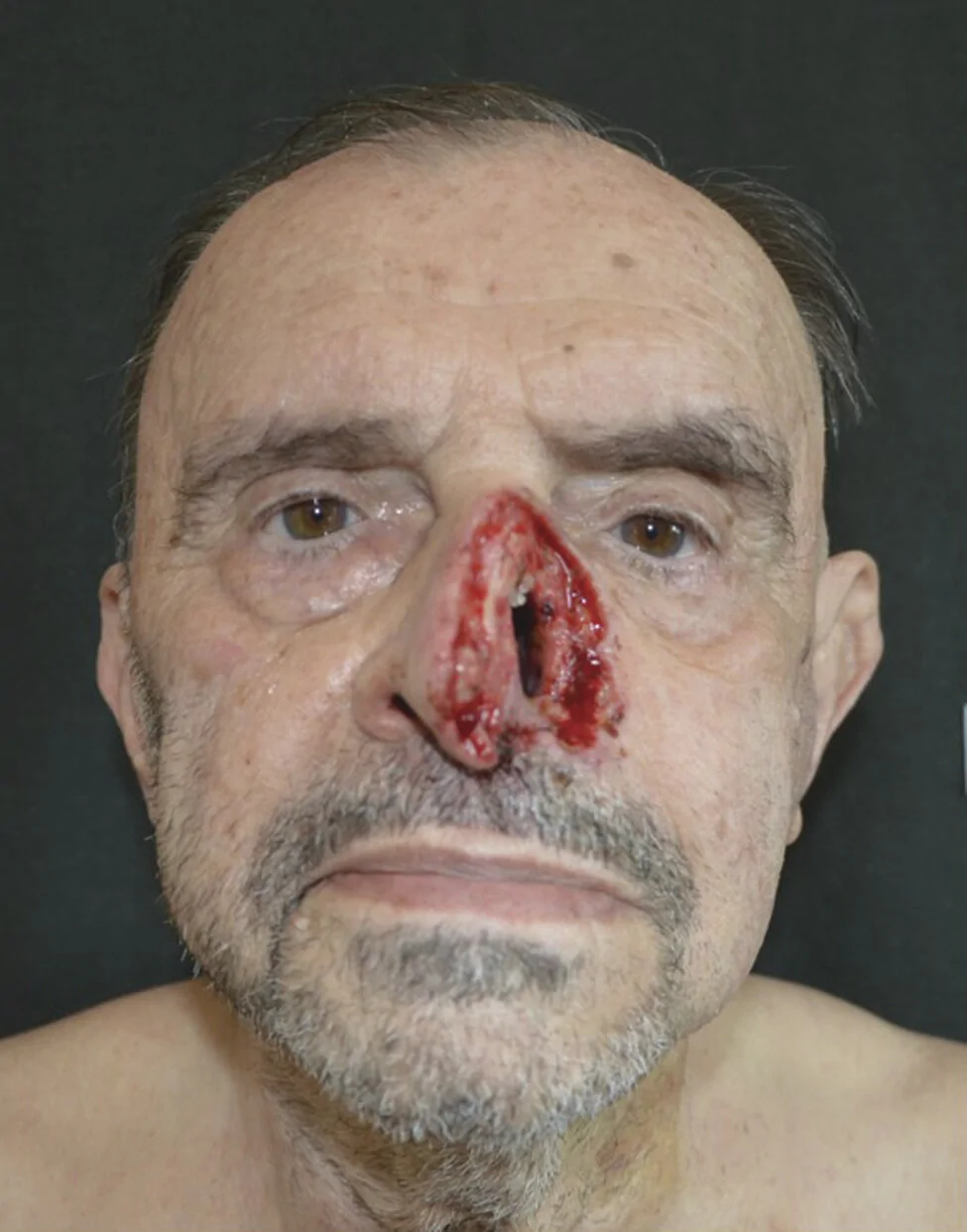
Preoperative appearance of the patient (front view).

Two templates of the missing lining and external skin were prepared preoperatively and transferred, respectively, onto the posterior auricular region (contralateral to the nose defect, for oncological safety in relation to lymph drainage pathways) and the homolateral forehead ( Figs. 3 and 4 ).

**Fig. 3:**
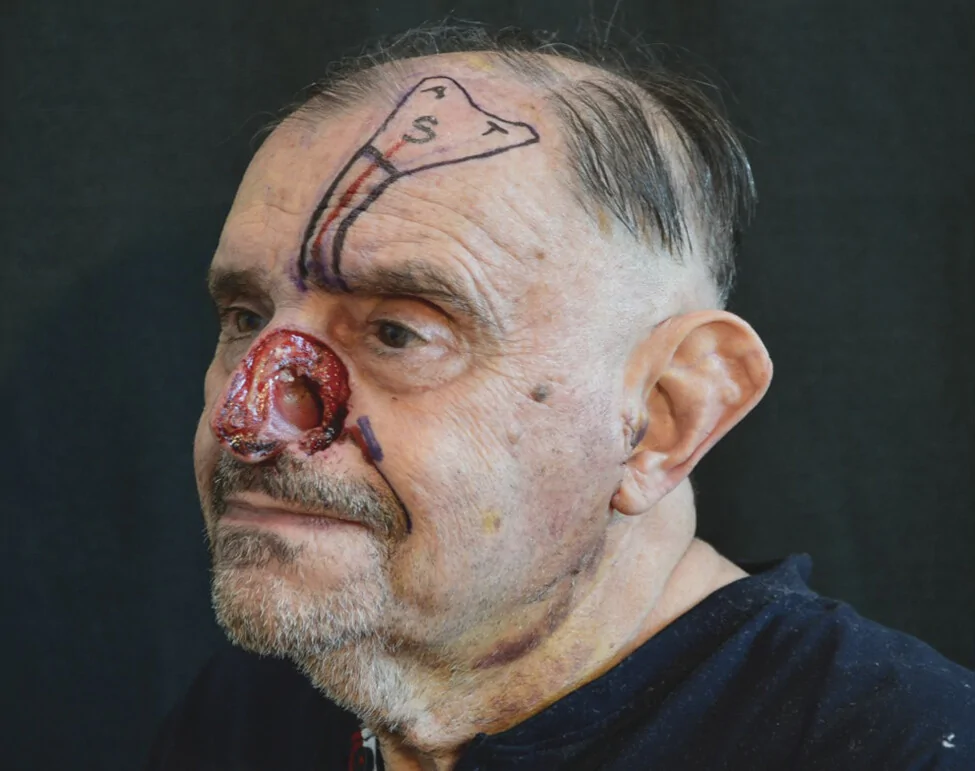
Preoperative markings on the auricular region.

**Fig. 4:**
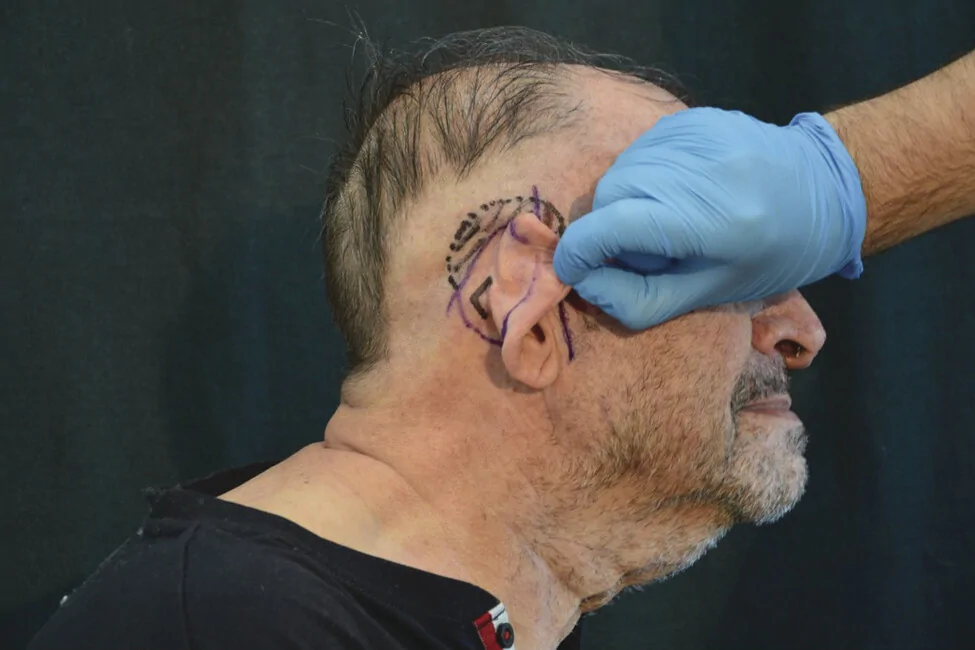
Preoperative markings on the frontal region.

A 7 × 4 cm TAPAS flap was elevated in a supra-perichondrial plane from the posterior auricular region, from distal to proximal, ending superiorly at the root of the helix, where the superior ramus originates from the superior temporal vessels (STVs). A cuff of adipofascial tissue was left around the superior branch of the superficial temporal artery, to allow the inclusion of venous branches to warrant an adequate outflow, and to keep anatomical variations. 19 Through a pretragal incision, pedicle dissection was performed until the exit from the parotid gland of the STV, obtaining a 4-cm pedicle ( Fig. 5 ), preserving both the temporal branch of the facial nerve and the temporal–auricular nerve.

**Fig. 5:**
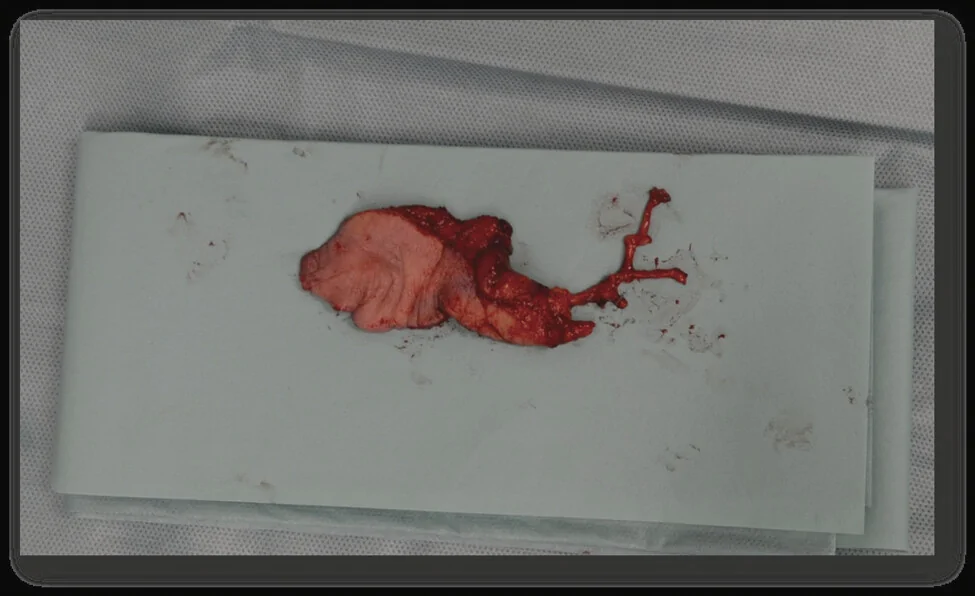
Image of a TAPAS flap raised with its pedicle. TAPAS, temporal artery posterior auricular skin.

Isolation of the left facial artery and vein was performed through an incision in the nasolabial groove as the recipient vessels.

The pedicle of TAPAS was divided and transferred to the hemi-nasal region and secured with interrupted stitches. Anastomoses were performed under a microscope with the facial artery and vein, in a termino-terminal fashion.

The skin of TAPAS was turned inside and inset to restore the hemi-nose and septal nasal lining.

Grafts of cartilage alone, without other surrounding tissues, were harvested from the base of the septum and another portion from the auricular concha, where the TAPAS flap was harvested, and then molded to recreate the nose framework.

Columellar and alar struts were created, and grafts were placed as supports for the external nose valve.

A forehead flap was then raised homolaterally to the defect with supra-periosteal dissection of the pedicle, and rotated clockwise to reach the left hemi-nose skin. TAPAS and forehead flaps undersurfaces created an envelope for the supporting cartilage.

The donor areas of the TAPAS and forehead flaps were directly closed ( Fig. 6 ). No infectious or vascular complications developed in the first 7 days. After 8 days, the patient was discharged and outplaced for 10 days after surgery.

**Fig. 6:**
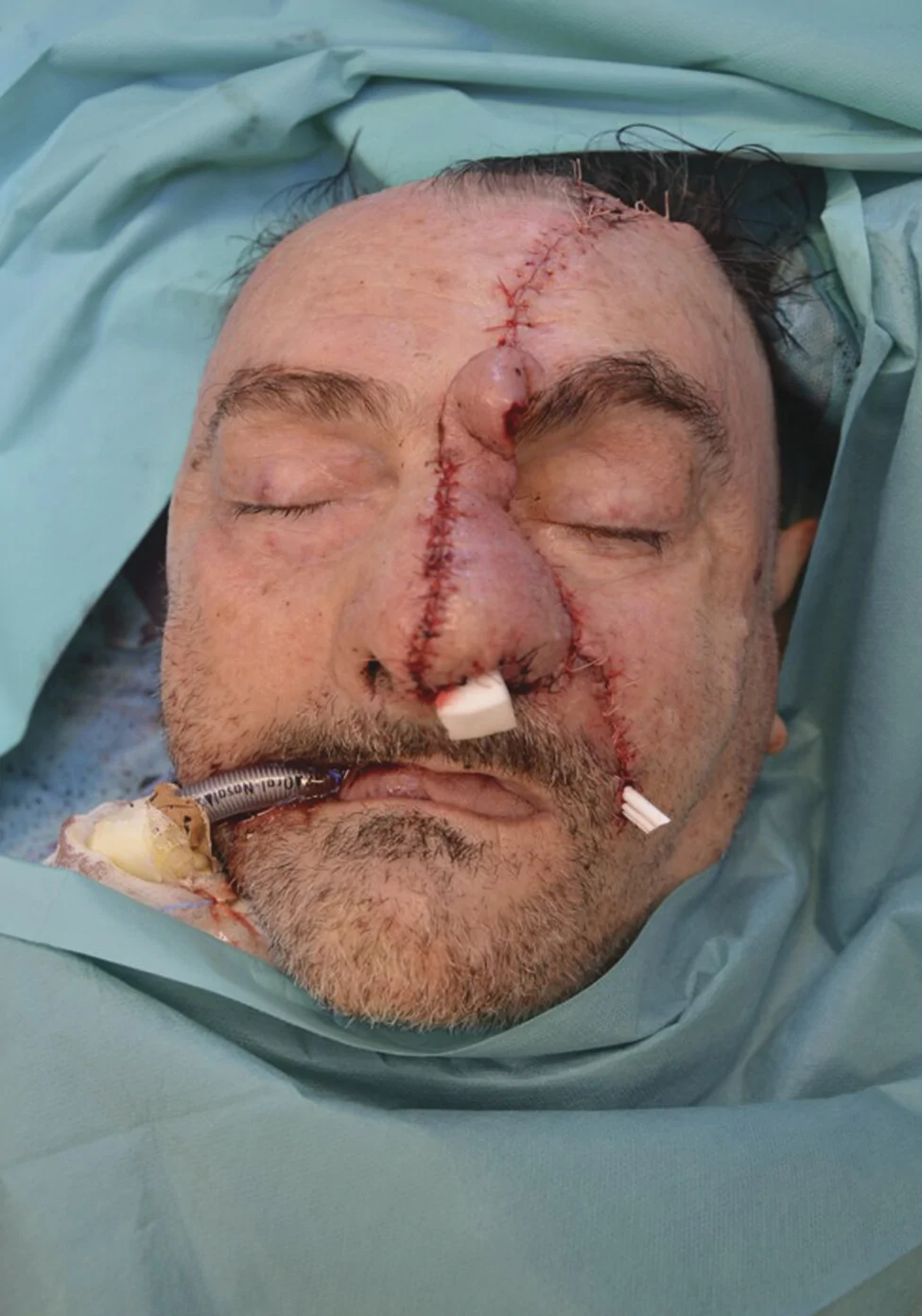
Final appearance after reconstruction of all three tissues.

After 4 weeks, a surgical intervention was performed to divide the pedicle of the forehead flap and to make minor skin paddle revisions. The patient received adjuvant chemotherapy with Ipilimumab. At 1 year of follow-up, the reconstructed nose is acceptable regarding color match and definition, with patent nostril and optimal airway flow, and no necessity for overnight splinting ( Figs. 7 and 8 ). The assessment of air flow was evaluated with a functional outcome assessment tool. The NOSE (Nasal Obstruction Symptoms Evaluation scale) is a simple score of five questions (each can be rated from 0 to 4, and the final result must be multiplied by 5) that revealed a good subjective perception of air flow, with an average score of 28 (range, 0–100). 20

**Fig. 7:**
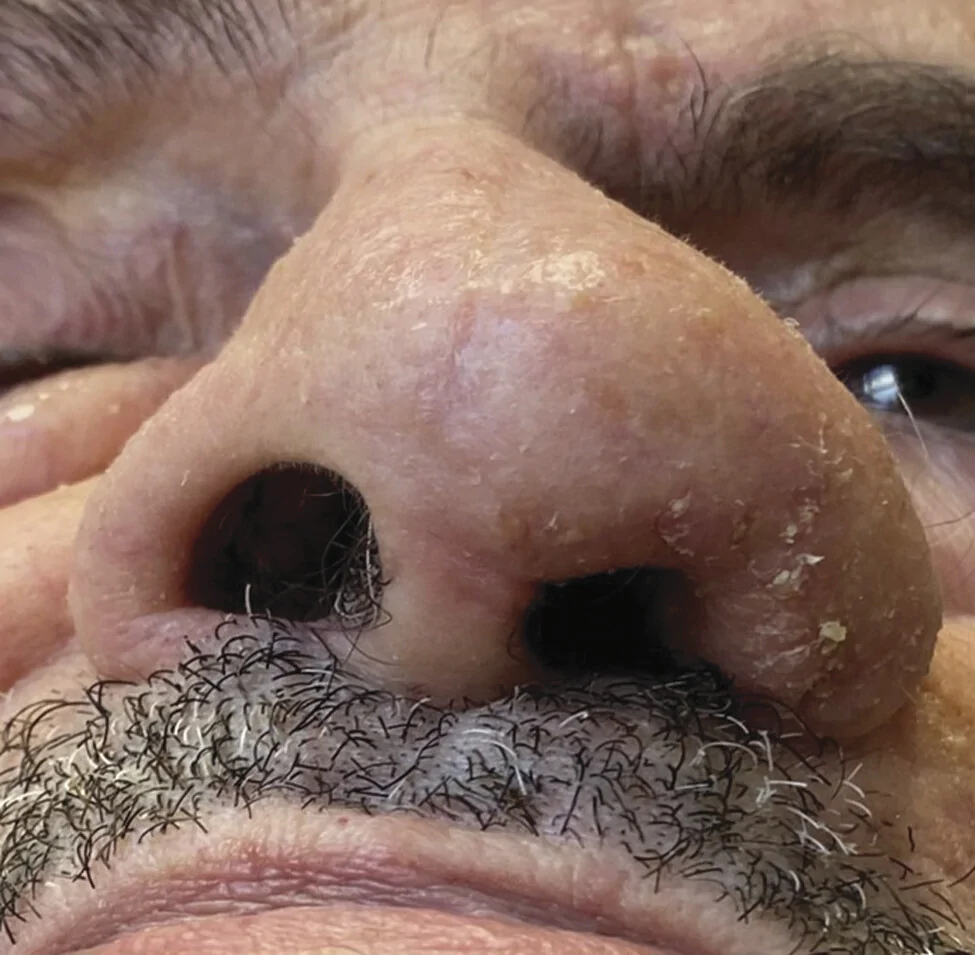
Appearance of the patient after follow-up period, from the bottom, revealing the thinness of the internal lining reconstruction with TAPAS flap. TAPAS, temporal artery posterior auricular skin.

**Fig. 8:**
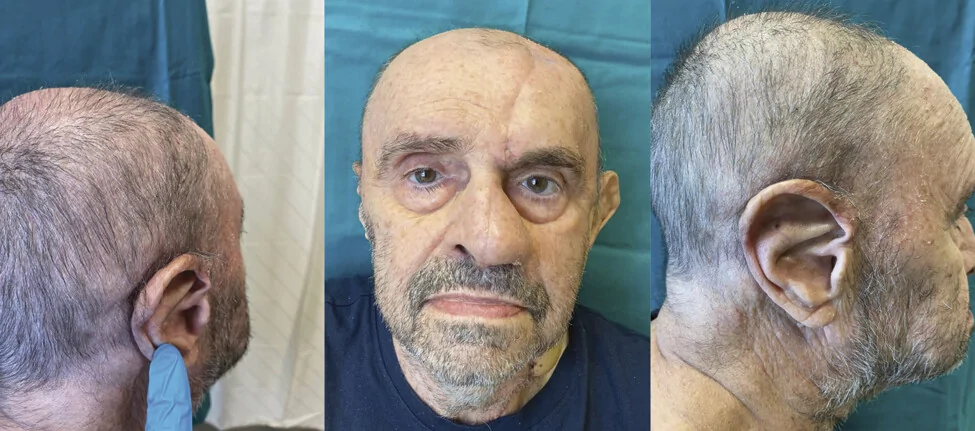
Anteroposterior appearance of the patient after the follow-up period, also with donor site demonstration.

After 16 months, the patient underwent a left parotidectomy and reconstruction with an anterolateral thigh (ALT) flap for a metastasis with skin infiltration.

## Discussion

Nasal reconstruction still represents a difficult procedure of reconstructive surgery for several reasons, with high demanding aesthetic function.

Reconstructive options in single-coanal defects of lining are scarcely reported in the scientific literature, and the discussion of the best technique is anything but defined, because of the necessity to balance the employment of local tissues or distant (pedicle or free) flaps.

Reconstructive options include a second forehead flap, but entailing a huge donor site defect and difficulty in folding the flap, as well as jeopardizing a backup procedure. Free flaps, which are usually “workhorses” for total or subtotal nasal reconstructions, appear to be a valid option, but with several drawbacks. 15 Indeed, microsurgery is technically challenging and time-consuming, while tissue harvested from distant donor sites rarely can provide a thin, pliable, and like-to-like skin or mucosa. 6

In this “no man's land” scenario, the usage of a flap harvested from the same surgical field (head and neck region) and with an inconspicuous donor site that can provide a good amount of high-quality tissue appears to be a valuable solution in hemi-nasal defects.

In the literature, based on the assessment of titles, abstracts, and full-texts, we found five articles reporting on the usage of the TAPAS flap. 16 17 18 19 21

We included all articles that employed it in every fashion and for the reconstruction of all parts of the body ( Table 1 ).

**Table 1:** Literature review table exposing the data available from the works reporting on the TAPAS flap

	Number of flaps	Max flap dimension (mm)	Maximum pedicle length (mm)	Number of flaps lost	Number of minor complications	Maximum follow-up (months)	Defect site	Donor site closure
Lassus and Lindford, 2017 16	7	25 × 10	59	1	1	20	Lower eyelid, oral cavity, lower lip, lateral nose	4 direct closure, 3 skin grafting
Lassus et al, 2018 17	4	70 × 60	60	0	2	14	Oral cavity, lower eyelid, lower lip	3 direct closure, 1 skin grafting
Ganry et al, 2020 19	2	60 × 50	Not reported	0	0	12	Lower eyelid, lateral nose	2 direct closure
Zena et al, 2024 21	20	95 × 60	60	1	6	17	Lower eyelid, nose, lower lip, oral cavity, palate, esophagus, larynx	18 direct closure, 2 skin grafting
Sahovaler et al, 2021 18	2	60 × 20	–	0	0	0	Lower gingivobuccal sulcus	Not reported

Abbreviation: TAPAS, temporal artery posterior auricular skin.

In total, only 35 cases are reported, but with a wide range of applications in head and neck reconstruction. Thanks to its intrinsic features, the TAPAS flap should be considered for wider employment in the future.

The maximum flap dimension that can be harvested is 95 × 60 mm, while the maximum pedicle length can be 60 mm. The most common indications for surgery were tumor resections in all head and neck regions (oral cavity, nose, eyelid, etc.). Other less common indications included esophagus fistula, mandible hardware exposure, and skin retractions of the eyelid.

Regarding surgical outcomes, flap complications were reported in 11 flaps (31.4%), which included total flap failure in 2 cases (5.7%), partial flap failure in 9 cases (37 %), and 1 case of hematoma (2.8 %). Donor site closure was performed with direct closure (eventually, with a part healed by secondary intention) in most cases.

Due to the paucity of articles found and of the case series, it was not possible to perform a meta-analysis or a statistical analysis with adequate reliability. For this reason, we decided to report and discuss the data themselves.

Given the statements previously discussed and the paucity of literature on the TAPAS flap, we present a new feasible option to reconstruct partial defects of the lining with tissues of excellent quality and an acceptable morbidity. Moreover, our technique spares other reconstructive tools, not jeopardizing backup options in the necessity of future salvage procedures.

TAPAS flap, in lining restoring, provides thin tissue (around 1–3 mm) with a constant vascular anatomy, and a negligible donor site morbidity and aesthetic impact (partial obliteration of the posterior auricular sulcus). Moreover, no important axial vessels are sacrificed in flap harvesting, which is different from other flaps ( Table 2 ). 4 14 15

**Table 2:** Advantages and disadvantages of free flaps used for lining reconstruction

Flap	Advantages	Disadvantages
Paramedian forehead flap	- Microsurgery not needed - Thin flap and skin	- The loss of one of the two flaps is the best reconstructive option for the external skin. - Unfavorable donor site cosmesis: If combined with a second paramedian forehead flap, it results in a very large forehead defect that may require a graft. - Limited amount of skin
TAPAS flap	- Very thin flap - Low donor site morbidity	- Limited amount of skin (7 × 4 cm maximum) - Short pedicle
ALT flap	- Large amount of skin - Low donor site morbidity - Long pedicle	- Thick flap and skin
Dorsalis pedis flap	- Thin flap and skin	- Donor site is often grafted with the underlying tendon and nerve structures - Unfavorable donor site cosmesis - Delayed donor site healing - Long-term and possibly permanent donor site symptoms - Sacrifice of a major vascular axis of the limb extremity
Radial forearm flap	- Thin flap and skin - Long pedicle	- Sacrifice of a major vascular axis of the limb extremity - Donor site is often grafted with the underlying tendon and nerve structures - Unfavorable donor site cosmesis

Abbreviations: ALT, anterolateral thigh; TAPAS, temporal artery posterior auricular skin.

Another advantage of our approach is the immediate reconstruction of both the external forehead flap and the internal lining; this combination gives the possibility to the surgeon to also restore the cartilaginous framework immediately, with a wide exposure of the surgical field. This flap does not require preliminary interventions to prepare the tissues that will be transferred to the nasal region, which is different from reconstructions that employ an expanded forehead flap. 6 15

A disadvantage of our surgical technique is the necessity of performing a microsurgical intervention, which is not always feasible. Another limitation is the amount of tissue that can be harvested. Furthermore, vessels have a small diameter (range: 0.5–1 mm) that matches the recipient vessels at a distal level (distal facial vessels or angular branch). 16 19

We can summarize that our technique restores at a single stage a whole half of the nose, combining microsurgery, cartilage grafting, and local flap raising, with a single surgical field and reducing the overall long nasal reconstruction period, according to Yen et al. 15

This article presents the first documented use of the free TAPAS flap for reconstructing the internal nasal lining in complex nose reconstruction, suggesting the need for further investigation and broader adoption in clinical practice.

## References

[1] Menick F J Salibian A Primary intranasal lining injury cause, deformities, and treatment plan Plast Reconstr Surg 2014 134 05 1045 1056 10.1097/PRS.0000000000000694 25347637

[2] Yen C I Yang J Y Chang C S Nose resurfacing with free fasciocutaneous flaps in burn patients Microsurgery 2018 38 06 659 666 10.1002/micr.30305 29427442

[3] Burget G C Walton R L Optimal use of microvascular free flaps, cartilage grafts, and a paramedian forehead flap for aesthetic reconstruction of the nose and adjacent facial units Plast Reconstr Surg 2007 120 05 1171 1207 10.1097/01.prs.0000254362.53706.91 17898591

[4] Hsiao Y C Huang J J Zelken J A The folded ulnar forearm flap for nasal reconstruction Plast Reconstr Surg 2016 137 02 630 635 10.1097/01.prs.0000475783.91456.3f 26818301

[5] Zenga J Chi J J Reconstruction of the intranasal lining Facial Plast Surg 2017 33 01 67 73 10.1055/s-0036-1597988 28226372

[6] Haack S Fischer H Gubisch W Lining in nasal reconstruction Facial Plast Surg 2014 30 03 287 299 10.1055/s-0034-1376874 24918708

[7] Weber S M Wang T D Options for internal lining in nasal reconstruction Facial Plast Surg Clin North Am 2011 19 01 163 173 10.1016/j.fsc.2010.10.005 21112518

[8] Menick F J The use of skin grafts for nasal lining Clin Plast Surg 2001 28 02 311 321,viii 11400825

[9] Sertel S Pasche P Pericranial flap for inner lining in nasal reconstruction Ann Plast Surg 2016 77 04 425 432 10.1097/SAP.0000000000000624 26418782

[10] Menick F J A new modified method for nasal lining: the Menick technique for folded lining J Surg Oncol 2006 94 06 509 514 10.1002/jso.20488 17061274

[11] Gasteratos K Spyropoulou G A Chaiyasate K Microvascular reconstruction of complex nasal defects: Case reports and review of the literature Plast Reconstr Surg Glob Open 2020 8 07 e3003 10.1097/GOX.0000000000003003 PMC7413796 32802686

[12] Walton R L Burget G C Beahm E K Microsurgical reconstruction of the nasal lining Plast Reconstr Surg 2005 115 07 1813 1829 10.1097/01.prs.0000164683.34767.2f 15923823

[13] Seth R Revenaugh P C Scharpf J Shipchandler T Z Fritz M A Free anterolateral thigh fascia lata flap for complex nasal lining defects JAMA Facial Plast Surg 2013 15 01 21 28 10.1001/2013.jamafacial.5 23108315

[14] Salibian A H Menick F J Talley J Microvascular reconstruction of the nose with the radial forearm flap: A 17-year experience in 47 patients Plast Reconstr Surg 2019 144 01 199 210 10.1097/PRS.0000000000005777 31246830

[15] Yen C I Kao H K Chang C S Simultaneous free flap and forehead flap for nasal reconstruction Microsurgery 2023 43 05 470 475 10.1002/micr.31020 36762616

[16] Lassus P Lindford A J Free temporal artery posterior auricular skin (TAPAS) flap: A new option in facial and intra-oral reconstruction Microsurgery 2017 37 06 525 530 10.1002/micr.30069 27159873

[17] Lassus P Husso A Vuola J More than just the helix: A series of free flaps from the ear Microsurgery 2018 38 06 611 620 10.1002/micr.30178 28370229

[18] Sahovaler A Berania I Ferrari M Temporal artery posterior auricular skin free flap for secondary oral cavity reconstruction Laryngoscope 2021 131 06 1297 1300 10.1002/lary.29250 33320348

[19] Ganry L Ettinger K S Rougier G Qassemyar Q Fernandes R P Revisiting the temporal artery posterior auricular skin flap with an anatomical basis stepwise pedicle dissection for use in targeted facial subunit reconstruction Head Neck 2020 42 11 3153 3160 10.1002/hed.26362 32686883

[20] Stewart M G Witsell D L Smith T L Weaver E M Yueh B Hannley M T Development and validation of the Nasal Obstruction Symptom Evaluation (NOSE) scale Otolaryngol Head Neck Surg 2004 130 02 157 163 10.1016/j.otohns.2003.09.016 14990910

[21] Zena M Homsy P Romanowski E Lindford A Lassus P Fifty free flaps from the ear J Plast Reconstr Aesthet Surg 2024 99 343 351 10.1016/j.bjps.2024.09.082 39418941

